# Observations on the Development of Special Senses

**Published:** 1882-04

**Authors:** John Dean Caton


### ART. VII.—OBSERVATIONS ON THE DEVELOPMENT OF SPECIAL SENSES.

BY JOHN DEAN CATON, LL.D.

It has been long observed that there is a wide difference among the different species of animals in the measure of sensibility in their various organs of sensation, and yet, so far as I know, the comparative physiologist has failed to detect sufficient reason for this difference in the structure of the organs.

In man the senses of seeing, hearing and of smell are all dull in comparison with many of the brute-creation, while the sense of feeling is probably more acute than it is in most of the lower orders. In many of the brute creation some of these organs are very acute and discriminating, while others are obtuse. Then, again, we may often observe a wide difference between individuals or varieties of the same species of animals in this regard. Take, for instance, the pointer and the setter-dog. Their sense of smell is very strong and discriminating, being able to distinguish different kinds of animals and birds by their odor alone and to determine the direction of their locality, at least approximately, while their sense of sight is dull. For instance, I have been led in the prairie a considerable distance by the dog up to a young grouse, which refused to rise till the dog was within a foot or two of the bird and in plain view, when the dog, being urged on, would spring forward to strike it with his front feet, but in a majority of cases would miss the bird by striking too far or not far enough or a little to one side, when if he had seen it he could not have failed to strike it. It may be said that long habit of the dog, having always depended upon the sense of smell and never upon sight, made him indifferent to all objects which came within the range of vision. Long habit of the active use of the sense of smell and the partial disuse of the sense of sight may have sharpened one and blunted the other, especially when this habit has been continued through many generations until its effects may have become hereditary. But can the physiologist detect any difference or any change in the structure of these organs which would tell him of the change?

Can he detect any difference in the organs of sight or of smell between the fox-hound and the grey-hound? The former pursues his game by the sense of smell alone, while the latter depends entirely upon the sense of sight. What difference does a critical examination disclose in the structure of these organs in the two varieties of the dog.

Thus far we have been dealing with an animal which has long been subjected to the dominion of man and to his modifying influences. Let us now turn for a moment to the wild animals. Take, for instance, the cervidea. Careful observations has shown that their sense of sight is very dull, while their senses of hearing and of smell are exceedingly sharp. These peculiarities apply to a whole race, and cannot be accounted for by any artificial means or peculiar surroundings. If the high senses of hearing and smelling are necessary, and so have been highly cultivated to enable them to avoid danger, why has the sight been neglected? Does the comparative physiologist find in the structure of these organs a satisfactory explanation for these extremes? Many other quadrupeds might be mentioned where wide differences in the sensitiveness of different organs are observed, but enough has been said to call attention to the subject.

We are not less interested when we study the habits of the birds. With these this is not a new subject or a new investigation. It has long been a question among careful observers whether a bird possesses the sense of smell—at least to a discriminating degree. The comparative physiologist unhesitatingly answers that they do, and many of them to a very high degree. He finds the organs of scent highly developed, and hence concludes *a priori* that they *must* possess the capacity to smell in a corresponding degree. But here the careful observer must be heard as well as the careful physiologist. It will not do to say that, if the facts dispute the theory, it is all the worse for the facts.

For myself, I have been a careful observer of facts tending to throw light on this subject, and have never yet found conclusive evidence that any bird possesses the power to smell. While many birds may possess that power, and that to a discriminating or even a high degree, I have failed to find one where opportunity offered which responded to the test. My opportunities for observing and testing nearly all of our game birds and water-fowl which are sought by sportsmen have been as good as those of most men, and I have tried to observe as carefully and impartially as I am capable of, and in no instance have I been able to detect any evidence that they could by the sense of smell alone detect the presense of an enemy under any circumstances. Many times have I lain carefully concealed, with a dog by my side, close to the edge of a water literally covered over with several species of ducks and geese, with the wind blowing from me to them, without being detected, even when some would come within a few feet of me. One such experiment had more interest to me than to have bagged all the birds upon the pond. A flock of ducks or geese will fly over and very near a man who is concealed without noticing him; but let them once get a glimpse of him, and they will sheer off in the greatest confusion.

My experience in this regard is not singular or exceptional. I never yet inquired of a sportsman who had not made the same observations, if he had thought of the matter at all. When approaching birds no one ever thinks of the course of the wind, while in stalking deer that is the first thing to be considered. With hunters of considerable experience this habit becomes intuitive, and is adopted almost without thought.

I have on two occasions had the same experience with pinnated grouse, where flocks of the birds, when seeking their food, have come very close to me, against the wind, without detecting me. Often I have lain concealed very close to a large flock of wood pigeons when feeding, with the wind blowing from me to them, without the least sign of detection being manifest. It is idle to say the birds were too busy to notice me if they did smell me, for the first glimpse of me by sight sent them away in haste.

The wild turkey, too, which has the reputation of having the keenest and most accurate sight of any living thing, seems to be quite destitute of the power of smell, although the comparative anatomist tells us that he has a good set of nasal organs, and should smell very well. I make nothing of my observations made in my acclimatization grounds at Ottawa, where I have kept them in large numbers for many years; but when hunting them in the woods I have had excellent opportunities for observation.

The wild turkey will approach the hunter in answer to his call from any direction, regardless of the course of the wind. He is a very suspicious and cautious bird, and may circle round the place whence he hears the call, trying to get sight of the hen that he thinks is calling him, but he never takes the alarm from smelling the hunter. If he can get the least glimpse of the smallest portion of his person, he is away instantly, or if he hears the least unusual noise or other suspicious circumstance; but never from smell.

I was once hunting on the Vermilion River, and saw at a considerable distance a large flock of wild turkeys cross the river and take a course directly towards me. I was at the foot of the bluff, near the top of which the underbrush, which clothed the side of the bluff and the bottom below, disappeared. I placed myself by the side of a tree, in a very dense thicket, with only my head above it. I soon heard the turkeys approaching, as I anticipated, distinguished by the constant *quit! quit! quit!* I stood perfectly still with the dog at my feet as the whole flock passed to the leeward of me, some of them but a few feet away, but beyond view. I saw their course would take them to the open near the top of the bluff. I stood without a motion, with the rifle at my face supported against the tree. At length, the head of the leading cock appeared, and instantly a loud note of alarm told his mates that I was discovered. That head and a few inches of the neck were alone visible for perhaps two seconds, when all took wing. Let me add, by way of parenthesis, that, before I had taken down the rifle, I heard a crash in the brush, and presently the largest common deer I ever killed, or ever saw, stopped opposite me in his rapid flight, not one hundred feet away. He had winded me, and stared directly at me for a few seconds and then bounded forward, and, after a few leaps, passed through an open space, when he fell to the earth never to rise again. Now, this deer detected my presence by its sense of smell when it was impossible to see me, for I could only partially see the outline of his form in the thicket, while the turkeys had passed me much nearer, and with an equally favorable wind, without giving the least evidence of a sense of smell; but the want of this sense was largely compensated by a quickness and accuracy of vision which I had not expected, familiar as I was with the habits of the bird. I could greatly multiply my own observations, all tending to show the feebleness or total want of the sense of smell in various species of the feathered tribes, and that the loss of this power is largely compensated by an increased power in other senses, but it would occupy more space than can be allowed me.

After all, this is a question of fact against theory; of observation and experience against what is claimed to be scientific deductions; for certain it is that the comparative physiologist has found the organs of smell in most species of birds, and in many in a very high degree. I am not able to state the exceptions, if there are any, as directly in point. I cannot forbear referring to the experiments of Mr. Audubon and Dr. Bachman upon the turkey-buzzard and the carrion-crow, as detailed in Audubon's American Ornithological Biography, vol. ii., p. 33, *et seq.* Some of these experiments may be briefly mentioned. Mr. Audubon stuffed a deerskin with hay, and when thoroughly dried, so as to emit no odor, laid it in a field, where a vulture attacked and worked at it and tore it till he exposed the contents, when, finding nothing he could eat, he left. He then took a dead hog, which he placed in a ravine, and covered it with vegetation, so it could not be seen, and there left it till so decomposed that it tainted the air to an intolerable degree for a long distance. The turkey-buzzards, which flew over and around the place and in the tainted atmosphere, did not discover it and paid no attention to it, although dogs were drawn to it by the sense of smell. He also had some young buzzards in a cage, which were ravenous at the sight of food, either fresh or offensive, but gave no signs of recognition when any kind of food was placed very near them which they could not see.

When these experiments were published, with the deduction that these birds find their food by the sense of sight rather than by that of smell, they were extensively and even harshly criticised in many quarters where it had been an adopted and even cherished theory that they are guided to their food principally or very largely by the sense of smell. Upon this Mr. Audubon requested Dr. Bachman, an eminent naturalist, to institute original and exhaustive experiments with the same birds in pursuit of the same inquiry. Dr. Bachman called to his aid a number of learned and scientific men, and they instituted a set of crucial experiments which were conclusive in the minds of all of them that these birds are led to their food by the sense of sight, and not by their sense of smell, and, it seems to me, must convince the impartial inquirer after truth that the sense of smell, if they have any at all, is so feeble as to be of no practical use to them in getting a living.

I may mention a few of these experiments. A quantity of offal and other animal matter were placed at the back of the garden, and a frame put around it so as to raise the brush with which it was covered a foot above the animal matter, leaving the sides open. This laid there for weeks, and became a very offensive mass without being discovered by the buzzards who were flying about it, and frequently very near it, through the tainted atmosphere. Then the most offensive portion of this matter was taken out and covered with a thin canvas cloth, on which pieces of fresh meat were placed. The birds lit upon the canvas and devoured the fresh meat. They never discovered the putrid and offensive mass beneath, although their bills were sometimes within an eighth of an inch of it. Then a small slit was made in the canvas, through which the birds discovered the food below, and at once attacked it. An experiment was tried with a blind bird. When food was placed in its mouth it was devoured with wonted eagerness; but when held within an inch of his nostrils no matter how offensive the food, or how hungry the bird, he did not notice it. These experiments with this blind bird were continued for twenty-four days, and always with the same result.

In order to determine whether these birds may be led to their food by the sense of sight alone, they made a coarse representation of a slaughtered sheep and exposed it in the field. The birds were directly attracted to it, and attacked it vigorously, and seemed greatly disgusted at the barren results.

Richard Owen, in his excellent work on Comparative Anatomy and Physiology, vol. ii., p. 132, gives our vulture very excellent olfactory organs, the nerve of which is five times as large as that of the wild turkey. He is so persuaded that these organs were made for use that he attempts to explain the two first experiments, above related by Audubon, consistently with an active sense of smell in the vulture; in the first, that the vulture, seeing the stuffed deerskin, thought he would try it, although he could smell nothing there, and in the case of the putrifying hog in the ravine, he concludes that, although the vultures smelt the carrion, not being burrowing animals, they would not venture upon an attempt to uncover it. We may regret that Mr. Owen did not favor us with his views upon Audubon's experiment with the young vultures, and especially of the experiments made by Dr. Bachman and several other eminent scientists, which were far more severe and conclusive, and seem quite incapable of explanation consistent with any power in those birds to distinguish anything by the sense of smell.

I have written this paper more to excite inquiry and investigation by those qualified to make them, than to establish a theory. The facts which are well established are that, in many animals, certain organs of sense are very dull, while others are remarkably acute. Is this explained by an examination of the organs themselves? Many birds, at least, have little capacity for smelling, if not entirely unable to do so; and, in that case, are the organs of smell dormant or nearly so, although present, and is it possible to detect any physiological cause for this? It seems to me that here is an interesting field for enlightened and impartial inquiry.

